# Sox2 overexpression alleviates noise-induced hearing loss by inhibiting inflammation-related hair cell apoptosis

**DOI:** 10.1186/s12974-022-02414-0

**Published:** 2022-02-28

**Authors:** Diyan Chen, Gaogan Jia, Yanping Zhang, Huanyu Mao, Liping Zhao, Wenyan Li, Yan Chen, Yusu Ni

**Affiliations:** 1 ENT Institute and Department of Otorhinolaryngology, Eye and ENT Hospital, Fudan University, Shanghai, 200031 China; 2 NHC Key Laboratory of Hearing Medicine (Fudan University), Shanghai, 200031 China

**Keywords:** Sox2, Cell protection, Hearing loss, Cochlear hair cells, Apoptosis, Inflammatory response, Lipopolysaccharide

## Abstract

**Background:**

The transcription factor Sox2 plays important roles in the developmental processes of multiple organs and tissues. However, whether Sox2 can protect mature or terminally differentiated cells against injury is still unknown.

**Methods:**

We investigated the roles of Sox2 in cochlear hair cells, which are terminally differentiated cells, using conditional transgenic mice and several hearing loss models.

**Results:**

Sox2 overexpression dramatically mitigated the degree of cochlear hair cell loss when exposed to ototoxic drugs. Noise-induced apoptosis of cochlear hair cells and hearing loss were also significantly alleviated by Sox2 overexpression. Notably, noise-induced upregulation of pro-inflammatory factors such as TNF-α and IL6 was inhibited by Sox2 overexpression. Then we used lipopolysaccharide to clarify the effect of Sox2 on cochlear inflammation, and Sox2 overexpression significantly inhibited lipopolysaccharide-induced upregulation of pro-inflammatory factors and alleviated inflammation-related cochlear hair cell death.

**Conclusions:**

These results demonstrate a novel protective role of Sox2 in mature and terminally differentiated cochlear hair cells by inhibiting inflammation.

**Supplementary Information:**

The online version contains supplementary material available at 10.1186/s12974-022-02414-0.

## Background

In mammals, cochlear mechanosensory hair cells (HCs) convert sound waves into electrical signals that are transmitted to the brain’s auditory centers to produce hearing. Aging, noise trauma, ototoxic drugs, and infections can lead to HC loss or degeneration and thus result in the onset of sensorineural hearing loss [1–7]. Because of the inability of HCs to regenerate after injury in the mature mammalian cochlea, protecting HCs against injury is extremely important for hearing preservation [8–10].

Sox2 (HMG-box transcription factor sex-determining region Y-box 2), a well-studied pluripotent transcription factor, is critical for the embryonic development of many organs [11–13]. For example, Sox2 serves as a regulator in the process of taste bud sensory cell differentiation from endodermal progenitor cells [12], and the expression of Sox2 has a strong influence on the branching process in the respiratory system [13]. Deficiency of Sox2 leads to abnormal cartilage development in the tracheal mesenchyme and to abnormal cellular composition [14], and Sox2 has been shown to play a significant role in the development of the inner ear [15]. In addition to its essential role in stem cell self-renewal and reprogramming, Sox2 also plays a role in the survival of stem cells and cancer cells [16]. Sox2 directly up-regulates the expression of Survivin, which inhibits the mitochondria-dependent apoptotic pathway in neural stem cells [17]. Although the roles of Sox2 in stem cells and cancer cells have been clarified in many studies, so far no studies about the possible role of Sox2 in mature or terminally differentiated cells have been reported.

In the development process of the inner ear, Sox2 is expressed in pro-sensory cells in the embryonic pro-sensory domain. When the terminal differentiation of cochlear progenitors is initiated, Sox2 expression is maintained in the supporting cells but gradually decreases in auditory HCs. Once the cochlea becomes fully mature and hearing function has been developed, the expression of Sox2 in HCs becomes undetectable [18, 19]. During the maturation of the vestibular sensory epithelium, Sox2 expression is maintained in supporting cells and type 2 vestibular HCs, but is gradually diminished in type 1 vestibular HCs. Sox2 is required for the maintenance of HCs during embryonic development of the zebrafish inner ear and for HC regeneration in zebrafish embryos after injury [20]. Our recent study reported that Sox2-negative type 1 vestibular HCs are more vulnerable than Sox2-positive type 2 vestibular HCs to 3,3'-iminodipropionitrile toxicity in adult mice [21], suggesting that Sox2 might exert a positive effect on the survival of sensory HCs. To explore the possibility that Sox2 might exert a positive effect on the survival of auditory HCs, we used conditional Sox2-upregulated transgenic mice and several cochlear HC injury models to investigate the possible roles of Sox2 in HC damage and survival.

## Materials and methods

### Animals and genotyping

The Rosa26-CAG-LSL (loxP-stop-loxP)-Sox2 transgenic mice, referred to as Sox2OE mice, were designed by our group and Beijing Biocytogen Co., Ltd. The Atoh1CreER and PrestinCreER transgenic mice, which have been described in detail in previous studies [22, 23], were the kind gifts of Prof. Zuo Jian. For the Atoh1CreER line, tamoxifen was injected subcutaneously once daily at 75 mg/kg body weight at postnatal day (P) 0–1. For the PrestinCreER line, tamoxifen was injected intraperitoneally once daily at 225 mg/kg body weight at P21–22. Mice of either sex were used for all experiments. Animals were bred under specific and opportunistic pathogen-free conditions, and tail DNA genotyping was performed for genetic identification. The primers used in the genotyping protocols are listed in Additional file 1: Table S2.

### Auditory brainstem response (ABR) measurement

Mice were injected with a solution of Zoletil (70 g/kg) and dexmedetomidine hydrochloride (1000 mg/kg) intraperitoneally to induce anesthesia. The efficacy of anesthesia was periodically ascertained through foot pinch response. The body temperature was maintained at 37 °C to avoid any influence on the auditory physiology test. ABR measurements were used to assess the mouse auditory function with a System 3 ABR workstation (Tucker Davis Technology, Alachua, FL, USA). Hearing function at 8, 16, 24, and 32 kHz was evaluated through an open field speaker, and acoustic thresholds of sound pressure level were determined using the BioSigRP software (Tucker Davis Technology, Alachua, FL, USA).

### Cochlear HC injury models

To construct the ototoxic drug-induced HC injury model in the neonatal stage, cochlear epithelia samples were obtained for explant culture from P2 transgenic mice that had received tamoxifen (Sigma, T5648-5G) administration at P 0–1. The tissues were treated with 1 mM neomycin (Sigma) for 6 h and then harvested after an additional 24 h in the absence of neomycin.

To construct the noise-induced HC injury model, the hearing function of P30 mice that had received tamoxifen administration at P21–P22 were examined by ABR test 24 h before noise exposure. The mice were then exposed to broadband noise at 116 dB for 2 h. Hearing function was evaluated again by ABR test 7 days after noise exposure, and the mice were killed and cochlear samples were harvested for morphological analysis.

To construct the lipopolysaccharide (LPS)-induced HC injury model, mice were given 10 μl LPS (Sigma-Aldrich, L2880) at 1, 2, or 4 mg/kg by tympanum injection and killed 3 days later.

### Immunofluorescence and morphological analysis

Immunofluorescence staining was performed as previously reported [32]. Rabbit polyclonal anti-Myosin7A (Proteus Biosciences, 1: 1000 dilution, 25-6790), goat polyclonal anti-SRY (sex-determining region Y)-box 2 (Sox2) (Santa Cruz Biotechnology, 1:1000 dilution, sc-17320), mouse monoclonal anti- Parvalbumin (Sigma, 1:1000 dilution, P3088), mouse monoclonal anti-Myosin7a (Santa Cruz Biotechnology, 1:500 dilution, sc-74516), rabbit mAb anti-TNF-α (Cell Signaling Technology, 1:400 dilution, 11948), and rat monoclonal anti-IL6 (ThermoFisher Scientific, 1:200 dilution, 11-7061-82) were used as primary antibodies, and control incubations were routinely processed without primary antibody treatments.

Propidium iodide (PI) staining was used to detect the apoptotic and necrotic HC nuclei after noise exposure as previously reported ^23^. Fixed cochleae were permeabilized in 1% Triton X-100 solution for 30 min and then stained with 5 mg/ml PI dissolved in PBS for 1 h at room temperature. After the final washes with PBS (10 min each), the tissue was dissected in PBS by removing the modiolus.

TUNEL staining was used to detect apoptotic HC death. Tissues were fixed with 4% PFA for 1 h, treated with 1% Triton X-100 37 °C for 40 s, and incubated in TUNEL staining solution (Roche) according to the manufacturer’s instructions.

### Confocal imaging and cell counting

Specimens were examined by confocal fluorescence microscopy (Leica SP8). To quantify the immunostaining-positive cells, nine separate segments along the entire cochlea were selected from the apex to the base.

### Real-time PCR

RNA was extracted from six cochleae of P30 adult mice from each group and was subsequently reverse transcribed into cDNA using Superscript III reverse transcriptase (Invitrogen) according to the manufacturer's instructions. Real-time PCR was performed on an ABI 7500 real-time PCR system (Applied Biosystems) using the TB Green™ PrimeScript™ RT-PCR Kit (Takara). The *Actb* gene was used as the endogenous control. Primer sets are listed in Additional file 1: Table S1. The quantification of relative gene expression, compared to *Actb*, was analyzed by the 2^−ΔΔCT^ method.

### Western blotting

Proteins were extracted from isolated cochlear epithelia with lysis buffer containing proteinase inhibitors, separated by SDS-PAGE gel, and transferred onto PVDF membranes (Immobilon-P; Millipore, Bedford, MA, USA). Membranes were blocked in 5% nonfat dried milk in TBST (20 mM Tris–HCl, 500 mM NaCl, and 0.1% Tween-20) for 1 h and later incubated with the primary antibodies overnight at 4 °C. The following antibodies were used for Western blot analysis: mouse monoclonal anti-beta-Actin (Thermo Fisher Scientific, 1:5000 dilution, MA515739), rabbit mAb anti-TNF-α (Cell Signaling Technology, 1:3000 dilution, 11948), and rat monoclonal anti-IL6 (ThermoFisher Scientific, 1:2000 dilution, 11-7061-82). The next day, the blots were washed three times with TBST and subsequently incubated with corresponding HRP-conjugated secondary antibodies for 1 h at room temperature. The immunoreactive bands were detected using an ECL kit (Pierce).

### Statistical analysis

Data were analyzed using GraphPad Prism 8. Student’s 2-tailed t-test was used to analyze the differences between two groups, and one-way ANOVA followed by the Bonferroni post-test was used for comparisons of differences among three or four groups. Data are presented as the means ± S.E.M, and *p* < 0.05 was considered significant.

### Study approval

This study followed the “Guiding Directive for Humane Treatment of Laboratory Animals” reporting guidelines enacted by the Chinese National Ministry of Science and Technology in 2006. All experiments were approved by the Shanghai Medical Experimental Animal Administrative Committee (Permit Number: 2009-0082) and were in accordance with animal welfare principles.

## Results

### Sox2 abundance is correlated with HC tolerance to ototoxic drugs in neonatal HCs

**Fig. 1:**
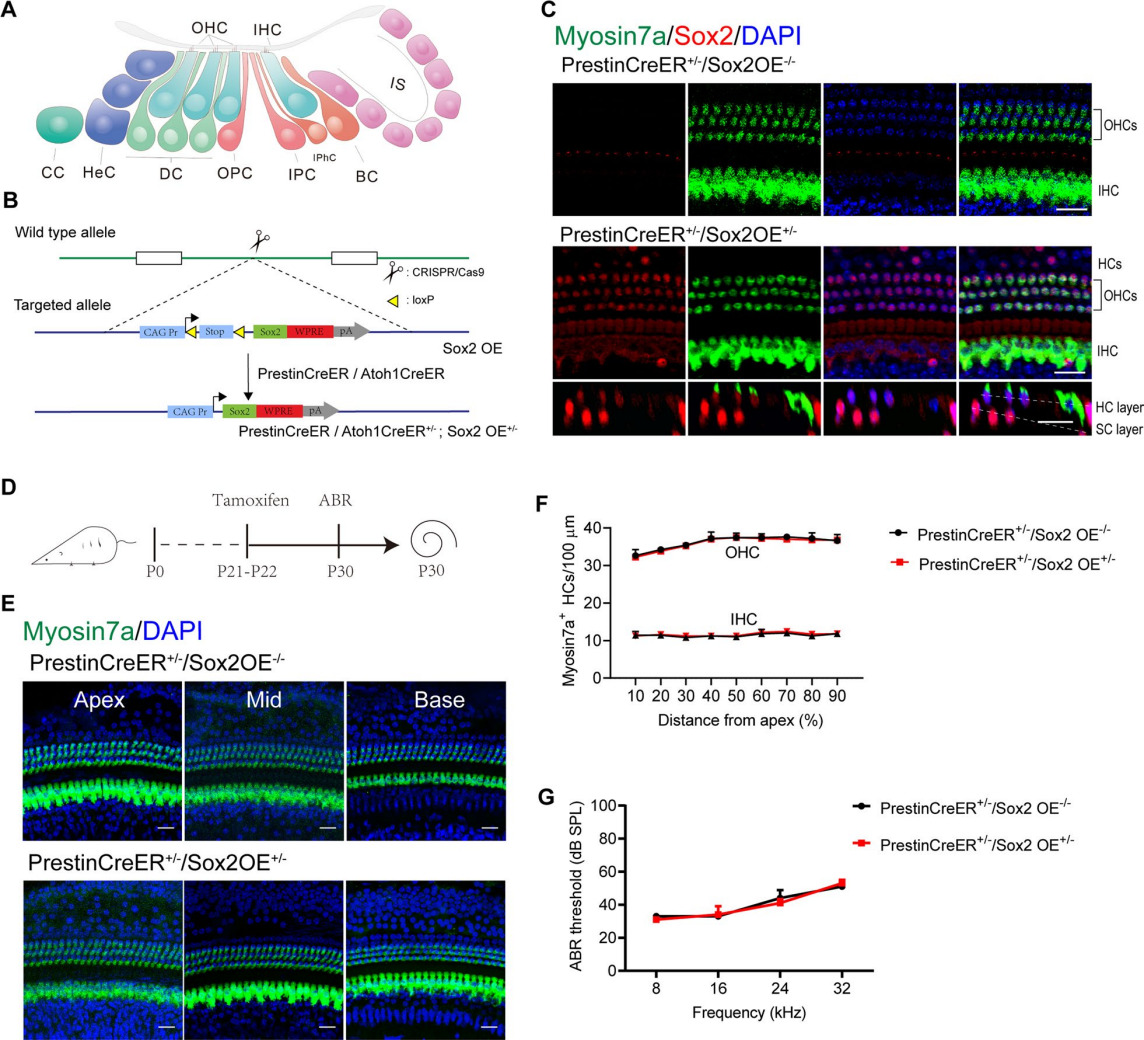
The cochlear morphology and hearing function were not affected by Sox2 overexpression. **A** The cross-section of the cochlear sensory epithelium. The IHCs and OHCs are located on the top layer, and most supporting cells (including BC, IphC, IPC, OPC, DC, and HeC) are located on the under layer. Some HCs can be observed in the top layer. *IPC* inner pillar cell, *OPC* outer pillar cell, *DC* Deiters’ cell, *IPhC* inner phalangeal cell, *BC* inner border cell, *HeC* Hensen’s cell, *IS* inner sulcus, *CC* Claudius cell. **B** Schematic for creating the transgenic mouse models. The Rosa26-CAG-LSL(loxP-stop-loxP)-Sox2 transgenic mice, referred to as the Sox2OE mice, were designed using the CRISPR/Cas9 method. **C** Images of Sox2 immunofluorescence (whole mount, HC layer) showed that there was no detectable Sox2 expression in the cochlear HCs of the control group, while in the PrestinCreER^+/–^/Sox2OE^+/–^ group Sox2 expression was clearly observed in OHCs. **D** Experimental protocol for E–F. Tamoxifen was injected once daily during P21–P22, and the cochlear morphology and hearing function were assessed by immunofluorescence and ABR at P30. **E** Representative Myosin7a immunofluorescence showed no difference in HC morphology between the PrestinCreER^+/–^/Sox2OE^+/–^ and control groups. **F** Quantification of IHCs and OHCs in the PrestinCreER^+/–^/Sox2OE^+/–^ and control groups. **G** The ABR thresholds of adult PrestinCreER^+/–^/Sox2OE^+/–^ and control mice. Data are presented as the mean ± SEM. *n* = 5 for each group. Scale bar = 20 µm

First, we examined the Sox2 expression pattern in the cochleae of wild type mice. Myosin7a was used as the marker of cochlear HCs. The cochlear epithelium is a delicate tissue containing region-specific HCs, which can be roughly classified into two categories, namely inner hair cells (IHCs) and outer hair cells (OHCs), which have different functions, gene expression patterns, and morphological characteristics [24] (Fig. 1A). Immunofluorescence results revealed that Sox2 was strongly expressed in all cochlear supporting cells, including inner border cells, inner phalangeal cells, inner pillar cells, outer pillar cells, Deiters’ cells, and Hensen’s cells (Fig. 1C). In the cochleae of adult control (PrestinCreER^+/−^/ Sox2OE^−/−^) mice, Sox2 was not observed in the cochlear HCs (Fig. 1C). However, in the cochleae of neonatal mice, Sox2 was also observed in some IHCs and OHCs, but the Sox2 abundance in HCs decreased from the apical to the basal turns (Additional file 1: Fig. S1A, note the Sox2 + HCs indicated by arrows in the apical turns). Similar to what was observed previously [25, 26], we found that the HC sensitivity to ototoxic drugs increased from the apical to basal turns because more HCs were lost in the basal turns compared to the apical turns in neonatal mice (Additional file 1: Fig. S1B).

### Sox2 overexpression in HCs did not alter HC morphology or auditory function under physiological conditions

To overexpress Sox2 in the cochlear HCs, we generated the inducible Rosa26-CAG-LSL-Sox2 mouse line in which forced expression of Sox2 was driven by the CAG promoter in a Cre-mediated manner. To examine the effects of Sox2 overexpression (Sox2OE) on cochlear HC survival in the neonatal and adult stages, respectively, we used two Cre mouse lines, namely, the Atoh1CreER line that targets the HCs including both IHCs and OHCs in the neonatal stage and the PrestinCreER line that targets OHCs in the adult stage (Fig. 1B). We generated PrestinCreER^+/–^/Sox2OE^+/–^ mice in which constitutive expression of Sox2 in Prestin^+^ cells were obtained by tamoxifen administration, and these are referred to as Prestin-Sox2OE mice. PrestinCreER^+/–^/Sox2OE^–/–^ mice served as controls. To evaluate the expression of Sox2 in OHCs and to determine the effect of Sox2OE on HC function under physiological conditions, Prestin-Sox2OE and control mice were injected intraperitoneally (i.p.) with tamoxifen from P21 to P22, and their hearing function and cochlear morphological features were assessed at P30 (Fig. 1D). In the control group, no Sox2 staining was observed in OHCs from mature cochleae, while nuclear staining of Sox2 was clearly observed in OHCs of Prestin-Sox2OE mice, confirming the successful induction of Cre activity and Sox2 overexpression in the Prestin^+^ cells (Fig. 1C). Immunofluorescence results showed that the morphological features and quantities of cochlear HCs in the Prestin-Sox2OE group were similar to those of controls (Fig. 1E, F). In addition, the ABR results showed that the hearing function of adult Prestin-Sox2OE mice was comparable to controls at 8, 16, 24, and 32 kHz (Fig. 1G). These results suggested that Sox2 overexpression in OHCs did not affect the maintenance of HC function.

### Sox2 overexpression protected HCs against aminoglycoside-induced injury in the neonatal stage

To explore the function of Sox2 in HC survival, we first used an in vitro aminoglycoside antibiotic injury model. We generated Atoh1CreER^+/–^/Sox2OE^+/–^ mice in which constitutive expression of Sox2 in Atoh1^+^ HCs was obtained by tamoxifen administration, and these are referred to as the Atoh1-Sox2OE group.

**Fig. 2:**
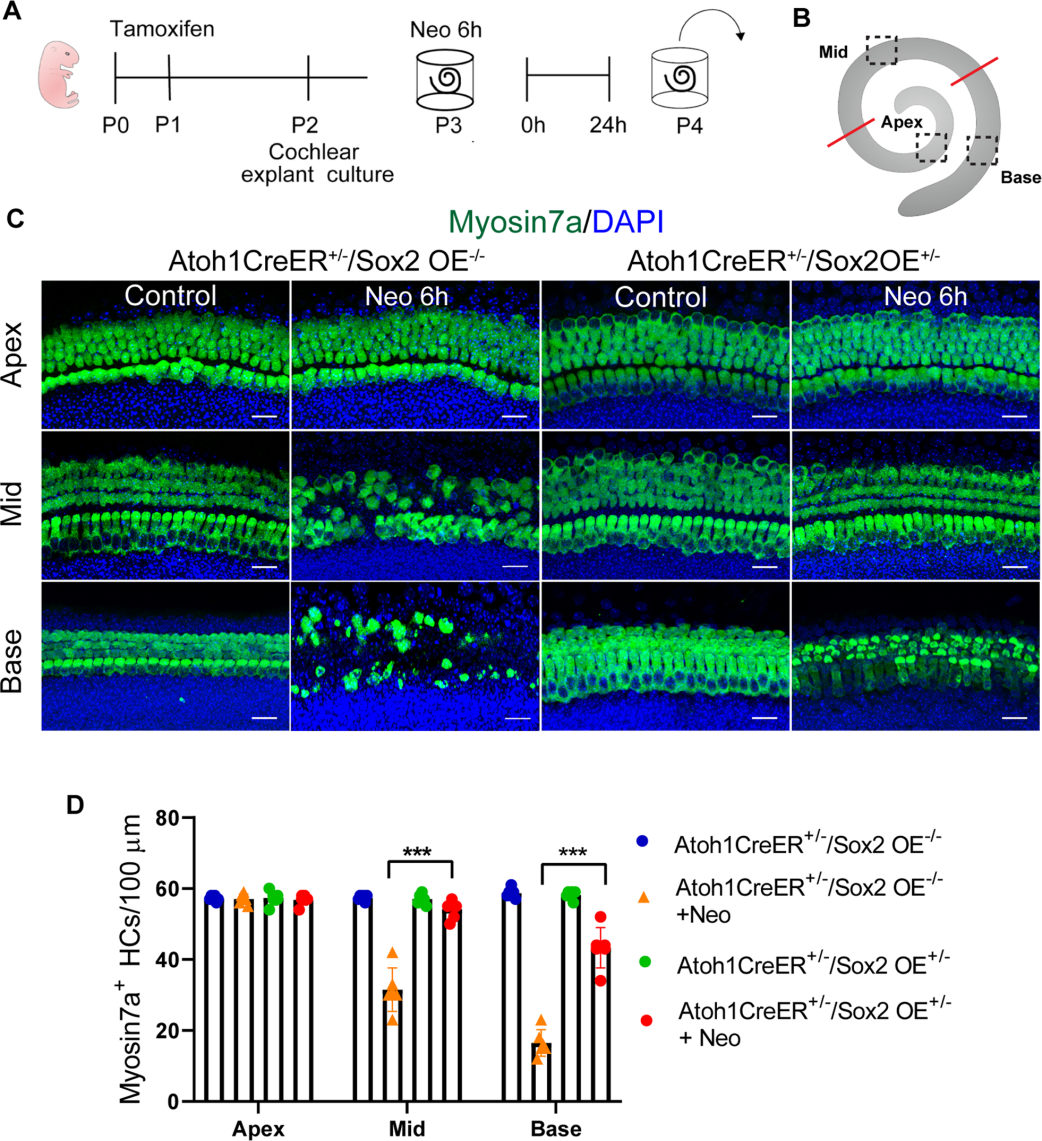
Sox2 overexpression protected HCs from aminoglycoside-induced injury in the neonatal stage **A** Experimental protocol for C-D. Cochlear epithelia from Atoh1CreER^+/–^/Sox2OE^+/–^ and control mice that received tamoxifen injection at P0–P1 were dissected and cultured at P2. Cochlear explants were treated with 1 mM neomycin for 6 h and harvested after an additional 24 h. **B** The scheme for cell counting. **C** Representative images of Myosin7a^+^ cells in Atoh1CreER^+/–^/Sox2OE^+/–^ and control mice treated with or without neomycin. **D** Quantification of the cochlear HCs per 100 µm in the Atoh1CreER^+/–^/Sox2OE^+/–^ and control groups. Data are presented as means ± SEM; *n* = 6 for each group; ***P < 0.001. Scale bar = 20 µm

The cochlear epithelium from P2 Atoh1-Sox2OE mice that received tamoxifen injections at P 0-1 were cultured and treated with 1 mM neomycin for 6 h and then allowed to recover without neomycin for an additional 24 h prior to harvesting (Fig. 2A). Atoh1CreER^+/–^/Sox2OE^–/–^ mice served as controls. Myosin7a immunofluorescence was used to determine the number of remaining HCs after neomycin treatment. In the control group, neomycin treatment led to massive HC loss, and the average numbers of remaining Myosin7a^+^ HCs were 57.0 ± 0.577, 31.5 ± 2.51, and 16.5 ± 1.52 cells/100 µm in the apical, middle, and basal turns, respectively (Fig. 2B–D). In the Atoh1-Sox2OE group, the average numbers of Myosin7a^+^ HCs were 56.8 ± 0.601, 54.2 ± 1.08, and 43.3 ± 2.33 cells/100 µm in the apical, middle, and basal turns, respectively. The average numbers of remaining HCs in the middle and basal turns from the Atoh1-Sox2OE groups were significantly larger than those from control groups (Fig. 2D, p < 0.001). Thus it appears that there is a protective effect of Sox2 overexpression against neomycin injury.

### Sox2 overexpression attenuated noise-induced HC damage and hearing loss in adult mice

**Fig. 3:**
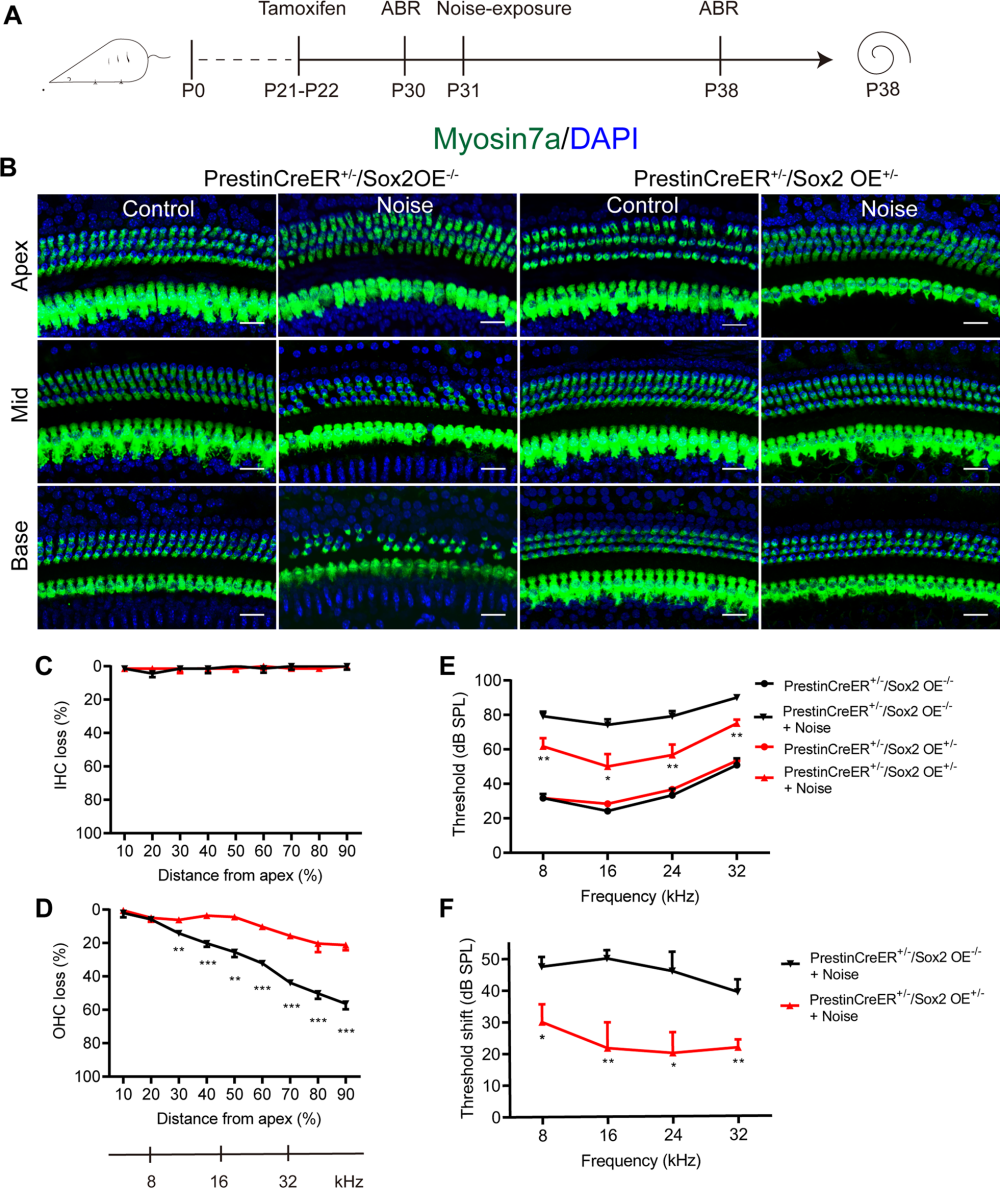
Sox2 overexpression alleviated noise-induced hearing loss and HC damage. **A** Experimental protocol for **B–F**. Adult PrestinCreER^+/–^/Sox2OE^+/–^ and control mice that received tamoxifen at P 21–22 were exposed to 116 dB broadband noise for 2 h. Cochlear morphology and hearing function were evaluated 7 days after noise exposure. **B** Representative images of Myosin7a^+^ cells in PrestinCreER^+/–^/Sox2OE^+/–^ and control mice treated with or without noise exposure. **C**, **D** Cochleograms showed noise-induced loss of IHCs and OHCs in the PrestinCreER^+/–^/Sox2OE^+/–^ and control groups. **E**, **F** Pure-tone ABR thresholds and threshold shifts in the PrestinCreER^+/–^/Sox2OE^+/–^ and control group 7 days after the noise exposure. Scale bar = 20 µm. Data are presented as means ± SEM; *n* = 6 for each group; *P < 0.05, **P < 0.01, ***P < 0.001, indicated the significant difference between the PrestinCreER^+/–^/Sox2OE^+/–^ with noise treatment and the PrestinCreER^+/–^/Sox2OE^–/–^ with noise treatment groups

To determine whether the susceptibility of HCs to noise exposure changed after Sox2 overexpression, P31 Prestin-Sox2OE mice that received tamoxifen at P21–22 were exposed to 116 dB broadband noise for 2 h (Fig. 3A). The hearing thresholds of the mice were determined using ABR tests at P38, and the cochlear specimens were harvested afterwards. PrestinCreER^+/–^/Sox2OE^–/–^ mice served as controls. In the control group, noise exposure led to significant OHC loss in the middle and basal turns of the cochlea (Fig. 3B–D). However, in the Prestin-Sox2OE cochlear epithelium, the noise-induced loss of OHCs was significantly attenuated in the middle and basal turns compared with the control mice (p < 0.01, Fig. 3B–D), indicating that Sox2-overexpressing cochlear OHCs were less sensitive to noise-induced injury.

Hearing thresholds before noise exposure (baseline values) showed no significant difference between the Prestin-Sox2OE and the control groups (data not shown). At 7 days after noise exposure, mice in the control group had significant hearing loss, as demonstrated by the increased hearing thresholds at 8, 16, 24, and 32 kHz (Fig. 3E). The noise-induced threshold shifts in Prestin-Sox2OE mice were significantly lower at all frequencies compared with those of the control mice (p < 0.05, Fig. 3F), suggesting that Sox2 overexpression in OHCs protects against noise-induced hearing loss.

### Sox2 overexpression protected OHCs by inhibiting the noise-induced apoptosis pathway

**Fig. 4:**
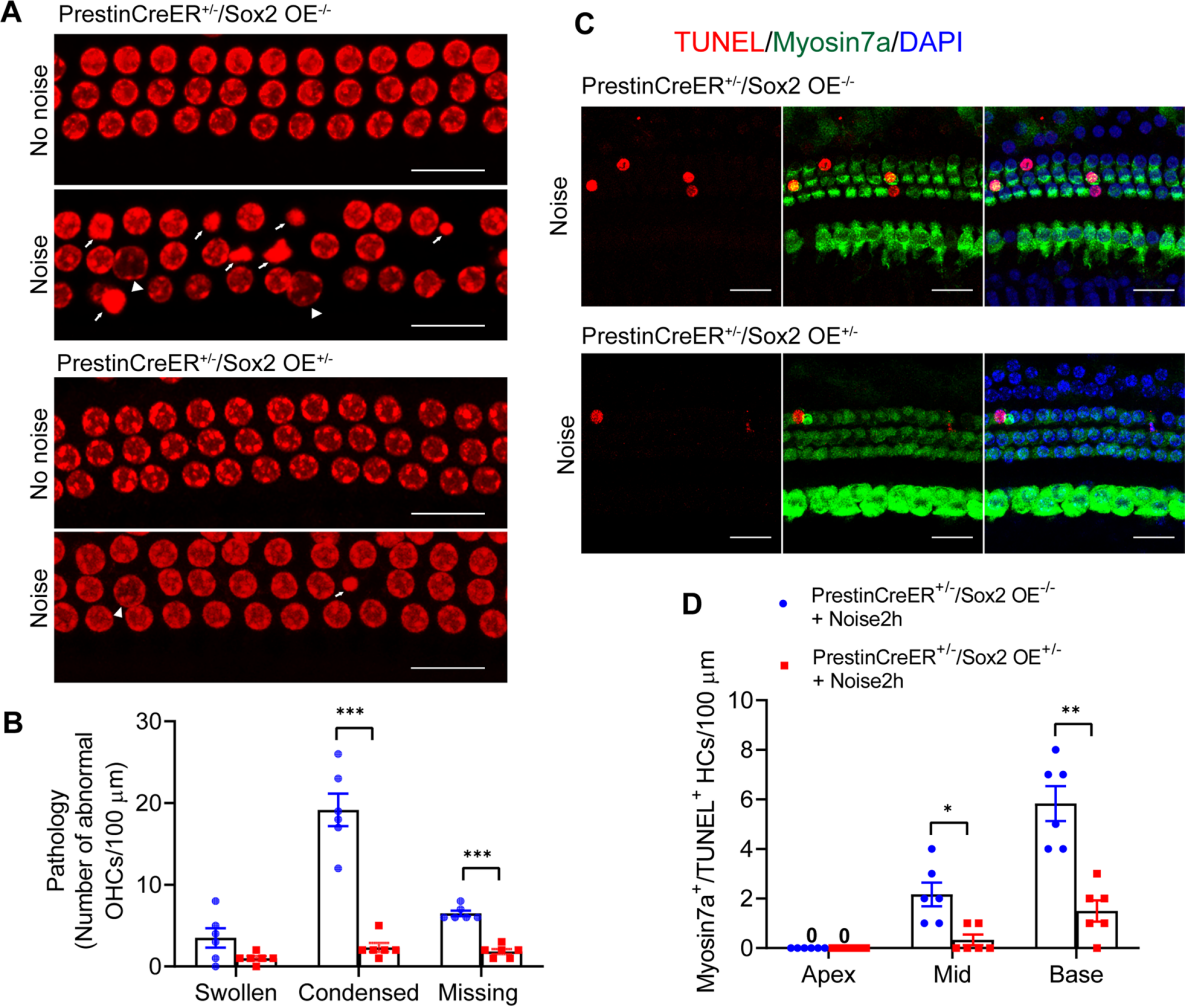
Sox2 overexpression suppressed noise-induced HC apoptosis. **A** Representative images of PI-stained OHC nuclei in the cochlear epithelium. Both swollen and condensed OHC nuclei were observed in mice treated with noise exposure. Arrowheads point to swollen necrotic nuclei, and arrows point to condensed apoptotic nuclei. **B** The numbers of swollen or condensed OHC nuclei and missing OHCs in the cochlear epithelium at 6 h after noise exposure. **C** Representative images of TUNEL staining and Myosin7a immunofluorescence of the cochlear epithelium. TUNEL^+^/Myosin7a^+^ OHCs were observed in the mouse cochlea at 6 h after noise exposure. **D** Quantification of TUNEL^+^/Myosin7a^+^ HCs in the cochlear epithelium. Data are presented as means ± SEM.; *n* = 6. *P < 0.05, **P < 0.01. Scale bar = 20 μm

To determine the mechanism through which Sox2 overexpression protects HCs against injury, we examined the apoptotic and necrotic changes in HCs after noise exposure using PI staining based on previous research showing the morphological changes that occur in OHCs after noise exposure [27]. In the control group, noise exposure induced morphological nuclear changes that included both apoptotic (condensed nuclei) and necrotic (swollen nuclei) changes in OHCs at 6 h after noise exposure (Fig. 4A, B). However, the number of apoptotic nuclei was significantly decreased in the Prestin-Sox2OE group when compared with the control group (Fig. 4A, B). Meanwhile, no significant differences in the numbers of necrotic nuclei were observed between the Prestin-Sox2OE and control groups. TUNEL staining was used to further determine the differences in apoptotic HCs between the two groups (Fig. 4C). Significantly fewer Myosin7a^+^/TUNEL^+^ cells were observed in Prestin-Sox2OE mice compared to control mice (Fig. 4D), suggesting that Sox2 overexpression suppressed noise-induced apoptosis of OHCs.

**Fig. 5:**
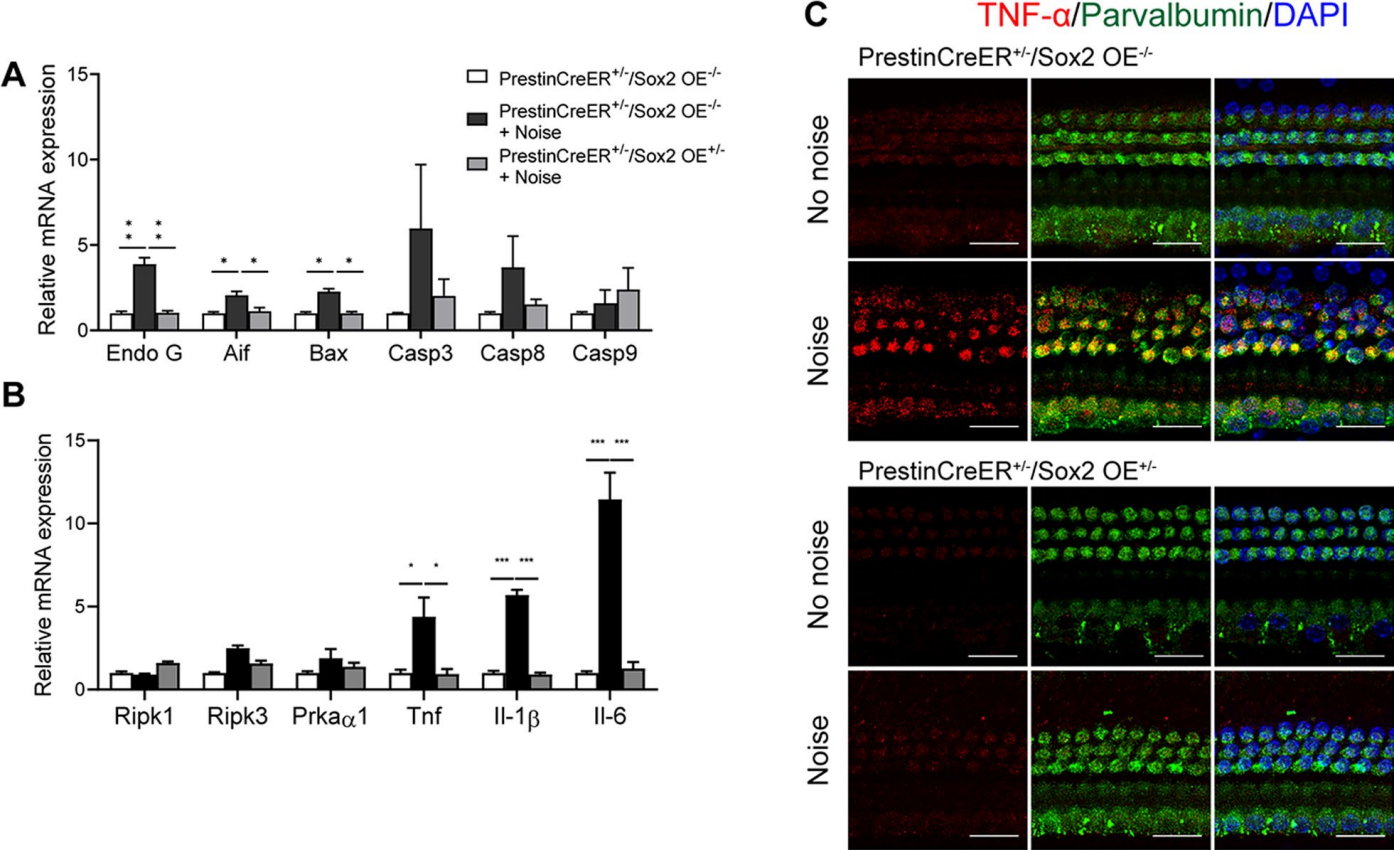
Sox2 overexpression suppressed noise-induced upregulation of inflammatory cytokines. **A** The mRNA expression levels of apoptosis-related genes (*Aif*, *Bax*, *EndoG*, *Casp3*, *Casp8*, and *Casp9*) were elevated 6 h after the completion of noise exposure. **B** The mRNA expression levels of necrosis-related genes (*Prkaα1*, *Ripk1*, and *Ripk3*) and pro-inflammatory cytokines (*Tnf*, *Il-1β*, and *Il-6*) were elevated 6 h after the completion of noise exposure. **C** TNF-α immunofluorescence in HCs after noise exposure. The TNF-α expression level was elevated after traumatic noise exposure in HCs, while it was inhibited in the Sox2 overexpression group. Parvalbumin is used as a marker of cochlear HCs. Data are presented as means ± SEM.; *n* = 6. *P < 0.05, **P < 0.01. Scale bar = 20 μm

We next assessed the relative mRNA expression level of apoptosis and necrosis-related genes at 6 h after noise exposure. In the control group, noise exposure induced the increased expression of both apoptosis-related genes (*Aif*, *EndoG*, and *Bax*) and necrosis-related genes (*RipK1* and *RipK3*) (Fig. 5A, B). In the Prestin-Sox2OE group, the expression levels of Aif, Bax, and EndoG were significantly decreased (Fig. 5A, p < 0.05), while the expression levels of RipK1 and RipK3 showed no significant difference when compared to the control group after noise exposure (Fig. 5B), further confirming that Sox2 overexpression protected OHCs by inhibiting the noise-induced apoptosis pathway.

### Sox2 overexpression alleviated HC damage by inhibiting inflammation

**Fig. 6:**
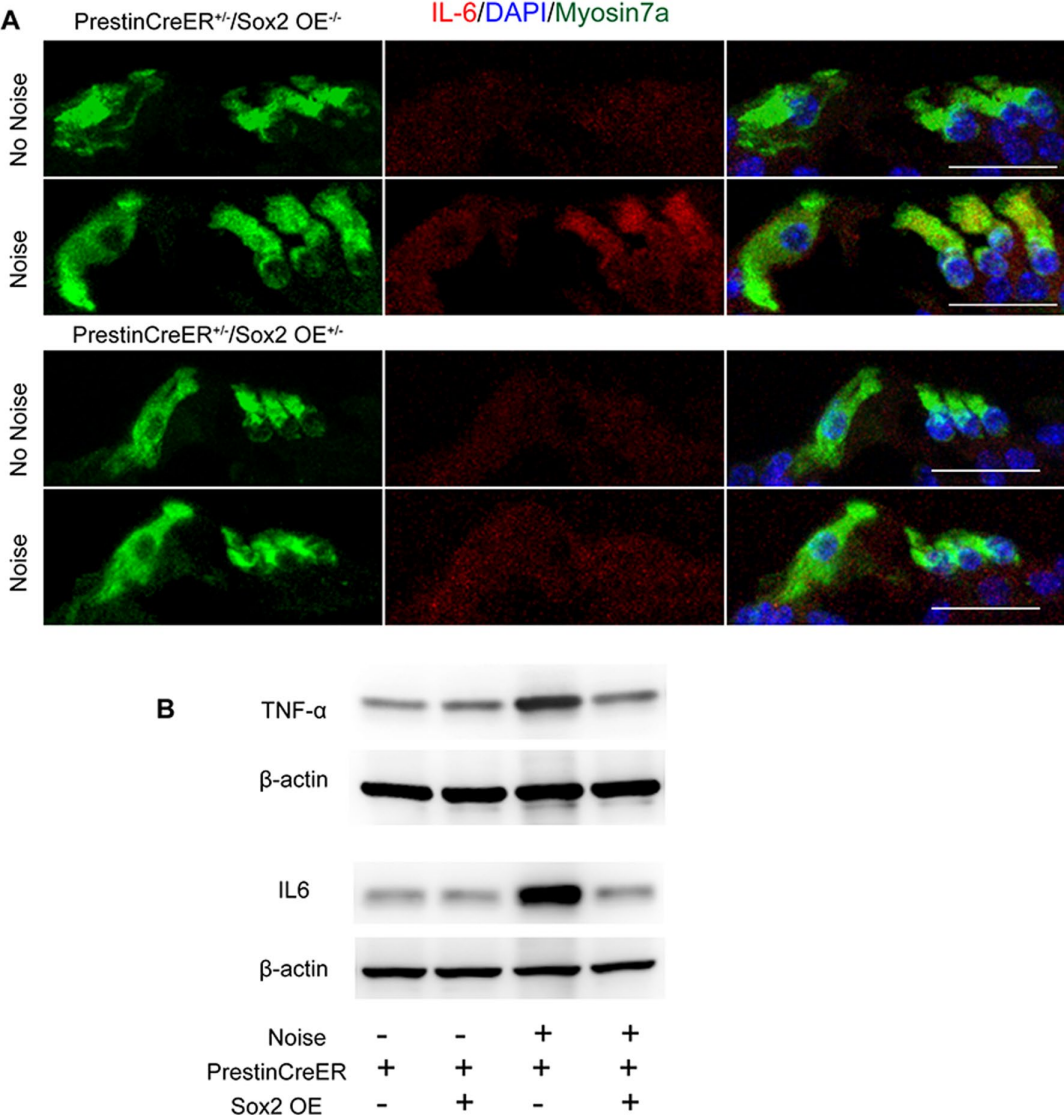
Sox2 overexpression suppressed noise-induced upregulation of IL6 and TNF-α. **A** IL6 immunofluorescence in cochlear cross-section after noise treatment. The IL6 expression level was elevated after traumatic noise exposure in HCs, while it was inhibited in the Sox2 overexpression group. Myosin7a is used as a marker of cochlear HCs. **B** Western blot analysis of sensory epithelia shows increased expression levels of TNF-α and IL6 in control mice after noise injury, but it was significantly inhibited in Prestin-Sox2OE mice. β-Actin served as the sample loading control. Data are presented as means ± SEM.; *n* = 3. *P < 0.05, **P < 0.01. Scale bar = 20 μm

In consideration of the important role of inflammation in HC death, the mRNA expression of pro-inflammation mediators was measured. In the control group, noise exposure induced significantly increased expression of *Tnf*, *Il-1β*, and *Il-6* at 6 h after noise exposure (Fig. 5B). In the Prestin-Sox2OE group, the expression level of these pro-inflammation mediators declined significantly to a level comparable to that in the group without noise exposure (Fig. 5B, p < 0.05*)*, indicating that Sox2 overexpression dramatically attenuated the noise-induced inflammation process. To further evaluate the protein expression level of pro-inflammatory mediators in cochlear epithelia, we performed Western blotting analysis of TNF-α and IL6. These results showed that noise exposure led to significant increases in TNF-α and IL6 protein levels in the control group, but Sox2 overexpression significantly inhibited the noise-induced increase of TNF-α and IL6 protein (Fig. 6). We also measured the expression of TNF-α and IL6 protein in the organ of Corti using anti-TNF-α and anti-IL6 antibodies, and we found that noise-induced TNF-α and IL6 expression in cochlear HCs was relatively lower in the Prestin-Sox2OE group compared to the control group (Figs. 5C, 6).

To determine the role of Sox2 upregulation in the inflammatory process, we constructed the lipopolysaccharide (LPS) challenge model. It has been previously reported that inflammation-related mRNA expression is upregulated in the cochlea within 6 h after LPS challenge [23]. To determine the optimal concentration of LPS in inflammation-mediated HC loss, 10 μl LPS at different concentrations (1 mg/kg, 2 mg/kg, and 4 mg/kg) was injected into the tympanic cavity of wild type mice and the cochleae were dissected for analysis 72 h later. LPS induced no obvious cochlear HC loss in the 1 mg/kg LPS group, mild HC loss in the basal turns in the 2 mg/kg LPS group, and severe HC loss in the middle and basal turns in the 4 mg/kg LPS group. Thus, 4 mg/kg of LPS was considered the optimal concentration for inducing significant HC loss in both the middle and basal turns.

**Fig. 7:**
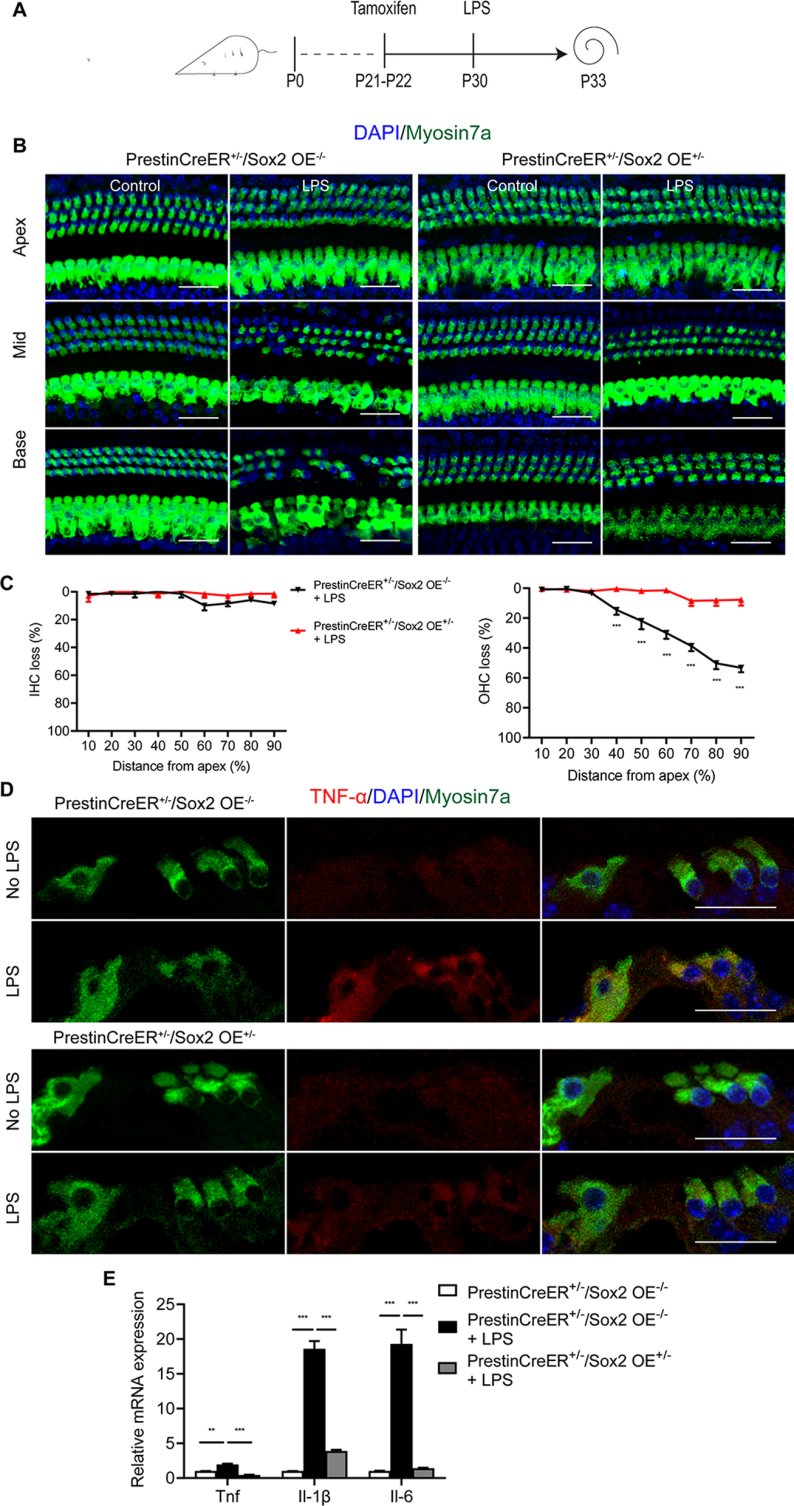
Sox2 upregulation mitigated inflammation-induced HC loss. **A** Experimental protocol for the LPS-induced HC loss model. **B** Myosin7a immunofluorescence in cochlear HCs of adult PrestinCreER^+/–^/Sox2OE^+/–^ mice and control mice with or without LPS treatment. **C** Cell counting results of cochlear HCs after 4 mg/kg of LPS administration. **D** TNF-α immunofluorescence in cochlear cross-section after LPS challenge. **E** The mRNA expression levels of pro-inflammatory cytokines. Sox2 overexpression prevented the LPS-induced increase in pro-inflammatory cytokines. Data are presented as means ± SEM; *n* = 6; **P < 0.01, and ***P < 0.001. Scale bar = 20 μm

**Fig. 8:**
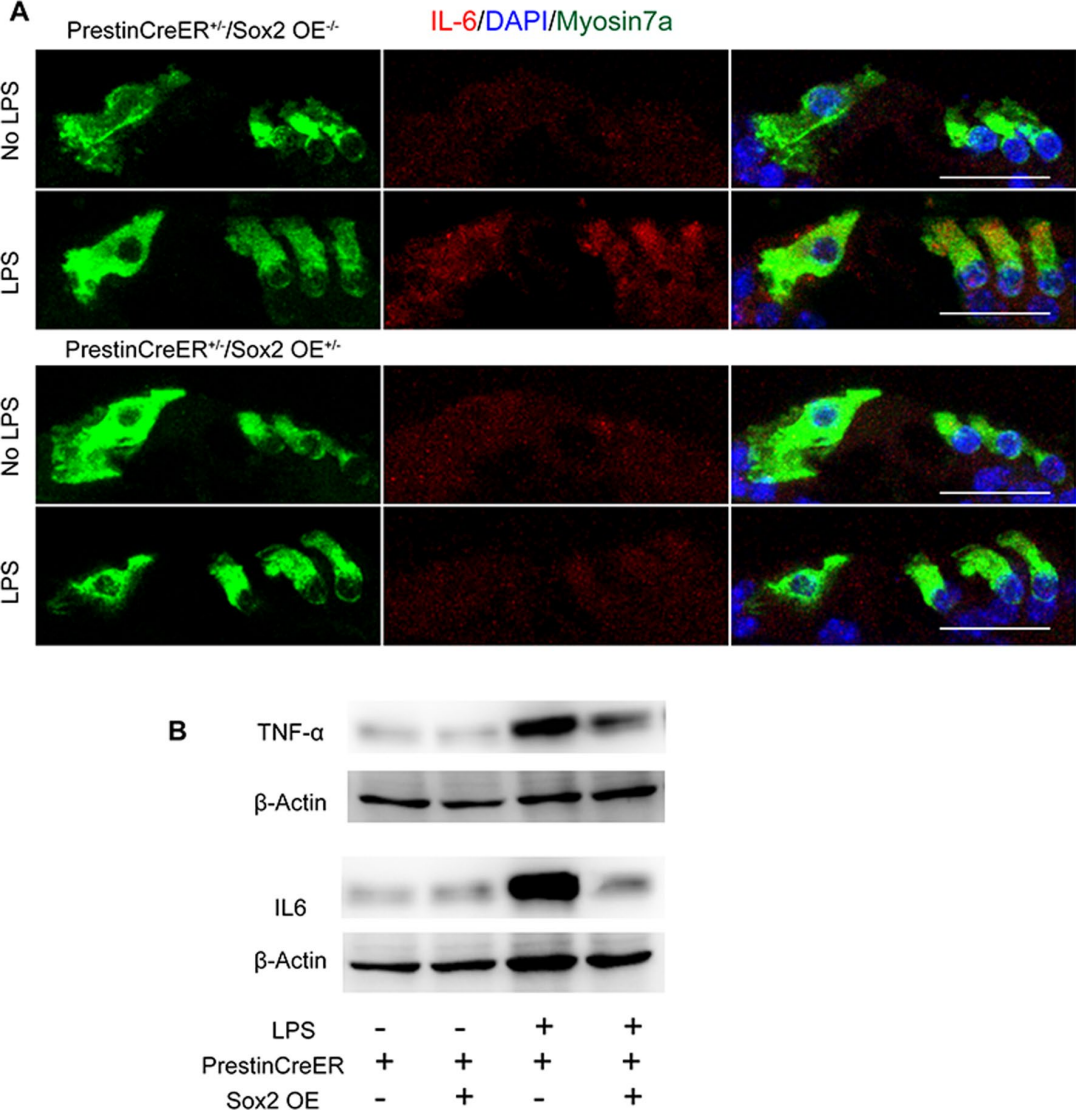
Sox2 overexpression suppressed LPS-induced upregulation of IL6 and TNF-α. **A** IL6 immunofluorescence in cochlear cross-section after LPS treatment. Myosin7a is used as a marker of cochlear HCs. (B) Western blot analysis of sensory epithelia shows increased expression levels of TNF-α and IL6 in control mice after LPS treatment, but the LPS-induced increase of the TNF-α and IL6 protein was significantly inhibited in Prestin-Sox2OE mice. β-Actin served as the sample loading control. Data are presented as means ± SEM.; *n* = 3. *P < 0.05, **P < 0.01. Scale bar = 20 μm

We compared LPS-induced HC damage between the Prestin-Sox2OE and control groups. Significantly more cochlear HCs were observed in the middle and basal turns in the Prestin-Sox2OE group than in the control group, indicating that Sox2 overexpression largely alleviated LPS-induced HC damage (Fig. 7A–C). We next evaluated the mRNA expression level of pro-inflammatory cytokines induced by LPS. qPCR results showed that Tnf, Il-6, and Il-1β increased substantially after LPS treatment in the control group, but their expression was relatively lower in the Prestin-Sox2OE group, which was a sign that the inflammation reaction was limited (Fig. 7E). Western blot analysis also showed very high expression levels of TNF-α and IL6 protein in the control mice and limited expression in the Prestin-Sox2OE mice after LPS treatment (Fig. 8). The immunofluorescence results also showed that the LPS-induced upregulation of TNF-α and IL6 in HCs was attenuated by Sox2 overexpression (Figs. 7D, 8). Collectively, these results showed that Sox2 upregulation inhibited the cochlear inflammatory response and subsequent HC death.

## Discussion

An estimated 466 million people are living with hearing loss worldwide. Although the exact mechanisms are not fully understood, oxidative stress, inflammation, autophagy, apoptosis, and necrosis have been reported to be involved in the onset and progression of sensorineural hearing loss [27, 28], and various strategies have been explored to try to protect sensory HCs and hearing. Some anti-apoptosis genes, like *Bcl2* and *Xiap*, have been reported to have protective roles in cochlear HCs and hearing [29, 30]. Antioxidants such as N-acetyl-L-cysteine, coenzyme Q10, and vitamin E have been reported to be effective in protecting against ototoxic drugs and noise exposure [31–34]. Some genes, like *Bmi1*, *G6PD*, *adenosine A1 receptor*, and *FoxG1*, protect HCs by inhibiting oxidative stress or by suppressing inflammatory pathways that are induced by ototoxic drugs or that are related to aging-related damage [28, 35–37]. Besides classic anti-apoptosis, anti-necrosis, and anti-inflammation factors, some development-related factors, such as Wnt/beta-catenin and Islet-1, have been shown to be capable of protecting HCs against injury and thus preserving hearing [35, 38].

In the cochlea, apical HCs are responsible for sensing sound at lower frequencies, and basal HCs are responsible for sensing sound at higher frequencies. Hearing loss in patients is generally more severe at higher frequencies than lower frequencies, and our experimental data showed that the sensitivity of HCs to injury increased from the apical to basal turns. The three different treatments—aminoglycosides (Fig. 2), broadband noise (Fig. 3), and LPS (Fig. 7)—all led to an apical-to-basal gradient of damage in cochlear HCs, and severe HC loss occurred in the basal turn while the mildest HC loss occurred in the apical turn. Previous studies showed that exposure to low-decibel broad band noise (8–16 kHz) results in HC damage mainly in the middle turns, but the HC damage extends to the basal turn when the intensity of the noise is greater than 100 dB [39–41]. The mechanism behind the difference in HC injury sensitivity along the cochlear turns remains unclear. In this work, we found that Sox2 was expressed in the neonatal cochlear HCs, which are less sensitive to injury (Additional file 1: Fig. S1), and Sox2 overexpression alleviated HC loss induced by aminoglycosides in the neonatal stage (Fig. 2), suggesting that deficiency of Sox2 in HCs in the basal turn might be one of the reasons for their vulnerability to ototoxic drugs.

The influence of noise exposure on people’s physiological and mental state has been the focus of increasing attention in the past decade [42, 43]. The impact of noise-induced hearing loss is broad and profound, including the loss of the ability to communicate with others and subsequent social isolation. Up to now, few medicines approved by the Food and Drug Administration have been confirmed to be effective in preventing or treating noise-induced hearing loss in clinical trials. Consistent with previous studies [44–46], our data further confirmed that apoptosis, necrosis, and inflammation are involved in noise-induced HC death (Figs. 4, 5). Aif and Endo G, which act as apoptotic mediators, translocate into the nucleus during HC injury and lead to HC apoptosis [47–49]. Prkaa1 (also known as AMPK) and receptor-interacting protein kinase (RipK) have been reported be associated with necrotic HC death [27, 50]. Although the endogenous expression of Sox2 is very low in mature cochlear HCs, forced Sox2 overexpression in Prestin^+^ OHCs has a strong protective effect against noise-induced hearing injury (Fig. 3). PI staining results showed that Sox2 upregulation decreased the number of HCs that underwent apoptosis (Fig. 4), and this was accompanied by a decrease in noise-induced expression of pro-apoptosis genes (Aif, EndoG, and Bax) and the number of TUNEL^+^ HCs (Fig. 5). In contrast, Sox2 overexpression had no obvious effect on the number of HCs that underwent necrosis, as demonstrated by the PI staining results and the mRNA expression levels of pro-necrosis factors (Figs. 4, 5). Together, these results suggest that Sox2 overexpression protects HCs and hearing against noise-induced injury by inhibiting HC apoptosis.

The inflammatory response activated by extrinsic attack and infection is also a strictly regulated process, and an appropriate inflammatory response can eliminate the pathogen, repair damaged tissue, and maintain cell homeostasis [51, 52]. However, dysregulation of the inflammatory response is pivotal in the initiation and progression of multiple diseases, such as age-related diseases [53] and neurodegeneration diseases [54]. In the auditory system, LPS can penetrate into the labyrinth through the round window membrane, where it stimulates the production and release of cytokines and mediates the initiation and development of inflammatory reactions in the media and labyrinth, which in turn leads to auditory epithelial injury and subsequent sensorineural hearing loss [55]. In our experiments, LPS administration caused a significant increase in the expression of pro-inflammatory factors and a significant loss of OHCs, but this inflammation-induced HC loss was alleviated by Sox2 overexpression (Fig. 7). Previous studies have shown that many pro-inflammatory cytokines and inflammatory mediators can lead to cell apoptosis [56–58]. For example, increased interleukin levels mediate cell apoptosis by regulating the Bax/Bcl-2 ratio in the cardiovascular system [56], and the TNF-α signaling pathway manipulates cell apoptosis through the Bax/Bak pathway or through a caspase-dependent pathway [57–61]. Our previous study showed that inhibition of the TNF-α pathway was an effective means of protecting HCs [26]. In this paper, we found that Sox2 overexpression dramatically suppressed LPS-induced and noise-induced upregulation of inflammatory cytokines and apoptosis-related genes (Figs. 5, 6, 7, 8), indicating that Sox2 overexpression inhibited the cascade of inflammatory cytokine release and subsequent inflammation-mediated HC apoptosis and hearing loss.

## Conclusions

We report for the first time that Sox2 protects mature and terminally differentiated cells against injury. Sox2 overexpression preserved hearing against noise exposure by inhibiting HC apoptosis, and this was correlated with the decreased expression of pro-inflammatory factors. These results thus suggest the protective role of Sox2 overexpression in hearing and sensory HCs and provide novel targets for strategies for the prevention and treatment of hearing loss.

## Supplementary Information

**Additional file 1: Table S1.** Primers used in qPCR. Table S2. Primers used in genotyping. Fig. S1. Cochlear HC sensitivity to ototoxic drugs increased from the apical to basal turns. (A) Images of cochlear cross-sections (orthogonal views) showing endogenous Sox2 expression in cochlear HCs and supporting cells in P2 mice. (B) Cochlear epithelia from P2 mice (wild type) were cultured and treated with 1 mM neomycin for 6 h, allowed to recover for 24 h, then fixed for Myosin7a immunofluorescence analysis. Scale bars: 20 μm.

## Data Availability

All data generated or analyzed during this study are included in this published article and its supplementary information files.

## Abbreviations

Aif: Apoptosis inducing factor
Endo G: Endonuclease G
Prkaa1: Phosphorylated-AMP kinase α
RIPK: Receptor interacting protein kinase
PARP-1: Poly(ADP-ribose) polymerase 1
ROS: Reactive oxygen species
Sox2: HMG-box transcription factor sex-determining region Y-box 2
TNF-α: Tumor necrosis factor
Il-6: Interleukin 6
Il-1β: Interleukin 1β
