# Supplementary material for: Sox2 overexpression alleviates noise-induced hearing loss by inhibiting inflammation-related hair cell apoptosis
Source: J Neuroinflammation. 2022 Feb 28;19:59. doi: 10.1186/s12974-022-02414-0 (PMC8883703; doi:10.1186/s12974-022-02414-0)
Supplement: Supplementary file 1 — Additional file 1: Table S1. Primers used in qPCR. Table S2. Primers used in genotyping. Fig. S1. Cochlear HC sensitivity to ototoxic drugs increased from the apical to basal turns. (A) Images of cochlear cross-sections (orthogonal views) showing endogenous Sox2 expression in cochlear HCs and supporting cells in P2 mice. (B) Cochlear epithelia from P2 mice (wild type) were cultured and treated with 1 mM neomycin for 6 h, allowed to recover for 24 h, then fixed for Myosin7a immunofluorescence analysis. Scale bars: 20 μm. [file 12974_2022_2414_MOESM1_ESM.pdf]

Supplementary information

Supplementary Table 1 Primers used in qPCR.

Gene	Forward Primers (5'-3')	Reverse Primers (5'-3')
Prkaα1	GTCAAAGCCGACCCAATGATA	CGTACACGCAAATAATAGGGGTT
Caspase 3	ATGGAGAACAACAAAACCTCAGT	TTGCTCCCATGTATGGTCTTTAC
Caspase 8	TGCTTGGACTACATCCCACAC	TGCAGTCTAGGAAGTTGACCA
Caspase 9	TCCTGGTACATCGAGACCTTG	AAGTCCCTTTCGCAGAAACAG
Ripk1	GAAGACAGACCTAGACAGCGG	CCAGTAGCTTCACCACTCGAC
Ripk3	TCTGTCAAGTTATGGCCTACTGG	GGAACACGACTCCGAACCC'
Tnf	GACGTGGAACTGGCAGAAGAG	TTGGTGGTTTGTGAGTGTGAG
Il-1β	GCAACTGTTCCTGAACTCAACT	ATCTTTTGGGGTCCGTCAACT'
Il-6	TAGTCCTTCCTACCCCAATTTCC	TTGGTCCTTAGCCACTCCTTC
Aif	TCCAGAGGCCGAAACAGAG	CATTTTGCCCCCTGATGAACC
Endo G	TTCCGCGAGGATGACTCTGT	CACCTGAGGCGCTACGTTG
Bax	TGAAGACAGGGGCCTTTTTG	AATTCGCCGGAGACACTCG
Actb	GGCTGTATTCCCCTCCATCG	CCAGTTGGTAACAATGCCATGT

Supplementary Table 2 Primers used in genotyping.

Genes	Primers (5'-3')
Sox2OE Wild Type F	AGTCGCTCTGAGTTGTTATCAG
Sox2OE Wild Type R	TGAGCATGTCTTTAATCTACCTCGATG
Sox2OE Mutant F	CCAGTAGACTGCACATGGCCC
Sox2OE Mutant R	GCCATACGGGAAGCAATAGCATG
PrestinCreER 1	CACAAGTTGTGAATGACCTC
PrestinCreER 2	TAACTGCTAGCATTTCCCTT
PrestinCreER 3	GTTAAAGAGCGTAATCTGGAACA
Atoh1CreER Mutant F	GCGGTCTGGCAGTAAAAACTATC
Atoh1CreER Mutant R	GTGAAACAGCATTGCTGTCATT

Supplementary Figure 1

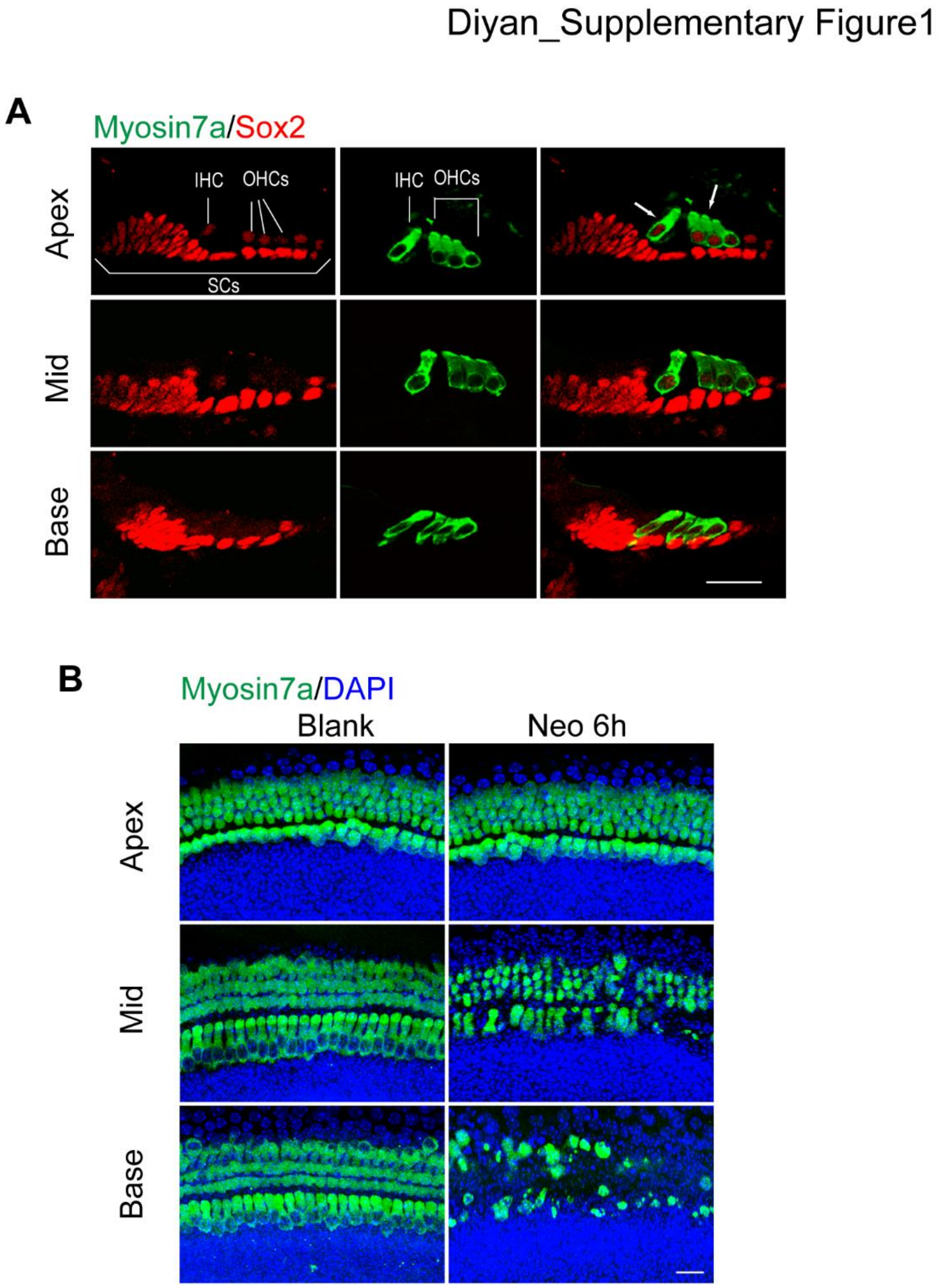

Figure S1

Cochlear HC sensitivity to ototoxic drugs increased from the apical to basal turns. **(A)** Images of cochlear cross-sections (orthogonal views) showing endogenous Sox2 expression in cochlear HCs and supporting cells in P2 mice. **(B)** Cochlear epithelia from P2 mice (wild type) were cultured and treated with 1 mM neomycin for 6 h, allowed to recover for 24 h, then fixed for Myosin7a immunofluorescence analysis. Scale bars: 20 μm.
